# Fan-lan Tai: a pioneer of plant pathology and mycology in China

**DOI:** 10.1093/procel/pwac031

**Published:** 2022-07-18

**Authors:** Yejing Ge

**Affiliations:** Department for the History of Science and Scientific Archaeology, University of Science and Technology of China, Hefei 230026, China

## Early studies in China and the United States

**Figure 1.**
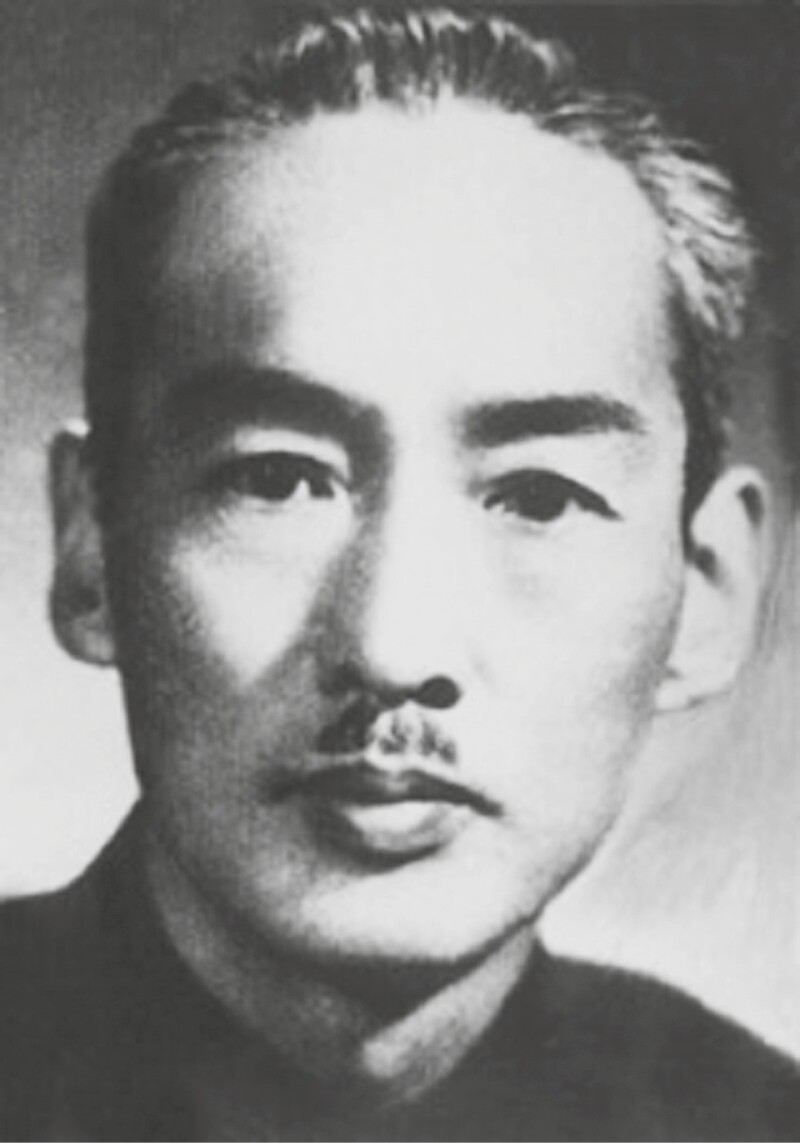
Fan-lan Tai (1893–1973).

Fan-lan Tai (戴芳澜, 1893–1973) was born in Jiangling, Hubei Province, to a dwindling family of bureaucratic landowners (Fig. 1). His grandfather, Hongxi Tai (戴洪禧), was a local township official in Longquan, Zhejiang Province. His father, Jing Tai (戴经), was a senior licentiate in the late Qing dynasty. His mother died when he was 8 years old. When he was 12, he attended the Higher Primary School affiliated with Nanyang Mission College in Shanghai. He withdrew from school for a year because of his family’s financial predicament. He was transferred to Wuchang Wenhua School, where he again withdrew the following year. In 1907, Fan-lan Tai and his brother entered Shanghai Aurora School, a missionary school with a focus on French teaching, laying a firm academic foundation and reinforcing his desire to continue studying.

In 1911, he passed the entrance exam at Tsinghua University and became one of the first international students of the American Boxer Indemnity. Domestic order was not yet restored when he finished his courses in 1913. Meanwhile, because the Boxer Indemnity was unavailable, he lingered for a year before embarking on official duty abroad (Lu et al., 1991). During this time, he returned to his hometown and worked as a substitute English teacher at a business school. In 1914, he went to the University of Wisconsin to study at the School of Agriculture. During that time, he became one of the first members of the Science Society of China (SSC) and served in the “solicitation committee” of the university, responsible for introducing new members. He transferred to the Department of Plant Pathology at Cornell University in 1916 and received a bachelor’s degree in agriculture 2 years later. During his college years, Fan-lan Tai published his earliest paper, “Speaking of Locusts,” in *Kexue* magazine (Tai, 1916). In a letter to his fiancée, he said, “After 4 years of college, my greatest feeling is the realization that I am ignorant! Learning is endless, and I will never graduate” (Ma, 1983). He then went to Columbia University, where he met two confidants, Robert Almer Harper (1862–1946), a pioneer in American fungal cytology, and Bernard Ogilvie Dodge (1872–1960), a mycologist and plant pathologist (Liu, 2008). However, Fan-lan Tai had to return to China because his father was unemployed and unable to support the family.

## A pioneer of plant pathology in China

In 1919, Fan-lan Tai received his first job as a professor at Nanjing First Agricultural College. He deliberately grew a small beard to look serious and older (Ma, 1983). Due to the chaos caused by factional disputes, he left the following year. During the summer vacation, he transitioned to gardening at a private farm in Tianjin. Soon after, he was employed as a professor at Guangdong Agricultural Special College, teaching botany and plant pathology, while also investigating diseases and insect pests in the agricultural experimental field. In 1921, he married Shuyuan Deng (邓淑媛). In Guangdong, he met Ying Ding (丁颖), a close friend with similar interests—later an academician at the Chinese Academy of Sciences (CAS). The most important contribution he made during his teaching in Guangdong was the discovery of Guangdong taro leaf blight in the summer of 1922. His paper “Taro Leaf Blight,” published in 1923, was the first research report on *Phytophthora* in China (Tai, 1923). Due to conflicts between warlords, soaring prices, and the frequent failure of the college to pay salaries, life was difficult.

In 1923, at the invitation of Bingwen Zou (邹秉文), a classmate at Cornell University, Fan-lan Tai became a professor of agricultural pests and diseases at National Southeast University. In addition to his lectures, he researched plant pathology and pathogenic fungi, usually going to the Institute of Biology of SSC to obtain data and complete research projects such as “Jiangsu wheat diseases and problems in Chinese plant pathology” (Wu et al., 2010). In 1927, “the university district” was reformed, and the original agricultural discipline system was abolished. Fan-lan Tai was dismissed again. In the same year, R.H. Porter—a professor who had held a course in plant pathology at the University of Nanking—returned to the USA and recruited Fan-lan Tai as a professor and director of the Department of Plant Pathology at the university of Nanking. In the 1920s, various scientific and technical societies and associations were established. He was keenly aware of the need to establish appropriate societies and organize academic exchanges to institutionalize phytopathology in modern China. In 1929, Bingwen Zou and Fan-lan Tai co-founded the Chinese Society for Plant Pathology (CSPP), and Fan-lan Tai succeeded as its president in 1936.

## A pioneer of mycology in China

Shortly after Fan-lan Tai entered the University of Nanking, Albert N. Steward (1897–1959), the head of the Department of Botany, informed him that the Institute of Botany at Harvard University had commissioned the collection of all Chinese fungal specimens. Fan-lan Tai requested that the collected specimens be sent in duplicate, with one specimen sent to the USA and a duplicate left in China. When Steward quipped that no one could identify fungal specimens in China, Fan-lan Tai replied, “No in the past does not mean no in the future! Let us start with this collection,” and thus began research on Erysiphales (Ma, 1983). In 1930, he published “A New Species of Erysiphales on *Acer buergerianum*,” which was the first new species of fungi studied and published in China using modern scientific methods, denoting the founding of Chinese mycology (Tai, 1930). From the middle of the 18th century to the first half of the 20th century, Westerners continuously collected fungal specimens in China. Fan-lan Tai was patriotic and attributed great importance to this, developing an article entitled “Collection of Fungi in China by Foreign Explorers.” In 1931, he published “Observations on the Development of *Myriangium bambusae* Rick,” which reported the detailed observation of the formation of the double-walled structure of Myriangiales, reflecting an approach to the study of fungal taxonomy that he developed—including the morphology and all biological traits such as cytology, developmental processes, and genetics. This article was recognized by the international mycological community and has been printed in a variety of authoritative mycology textbooks. *Introductory Mycology*, an internationally recognized authoritative textbook, still cited this work until 1994.

In 1934, Tsinghua University established the Institute of Agriculture and proposed appointing Fan-lan Tai as the director of the Plant Pathology Laboratory. At the same time, Dafu Yu (俞大绂) returned from his studies in the USA and took over Tai’s work at the University of Nanking. At that time, Fan-lan Tai had also applied for a research grant from the Education and Culture Foundation of China to study fungal genetics at the New York Botanical Garden and Cornell University (Wu et al., 2010). During his visit, he collaborated with Dodge on the taxonomy and cytogenetics of *Neurospora*, and his research papers, “Two new species of *Neurospora*” and “Sex-reaction Linkage in *Neurospora*,” were published in *Mycologia* (Qing, 2006). At that time, he continued to follow the preparatory work of the Plant Pathology Group at Tsinghua University by correspondence.

During the War of Resistance against Japanese Aggression (1931–1945), Tsinghua University was forced to move south, and the Institute of Agriculture was relocated to the village of Dapuji in the western suburbs of Kunming. During these hard times, Fan-lan Tai went to the city once a week to give lectures at the National Southwest Associated University and spent the rest of the time doing research. To continue their projects, Tai and his students started a farm. Taking advantage of the natural ecological conditions in Kunming, he conducted in-depth research on the abundant local species of *Geoglossum sinense* in Yunnan and discovered a new rice disease, *Ephelis oryzae*, in a makeshift laboratory (Mycosystema, 1983; Liu, 2020). After war was won, Tsinghua University moved back to Beijing and the Institute of Agriculture was expanded to the School of Agriculture, with Fan-lan Tai becoming the director of the Department of Plant Pathology. He brought back all the scientific materials from Kunming, and nearly 20 papers published by himself and his colleagues from 1945 to 1948 were the result of successive research materials brought from Kunming to Beijing. He prioritized resuming his teaching and research work as soon as possible, intensified the preparation of teaching materials, prepared to start classes, convened a meeting of professors to discuss research plans, and finished the Kunming work late at night. In 1947 and 1948, he published “Uredinales of Western China” and “Cercosporae of China II.” In 1948, Fan-lan Tai was elected as an academician in the Biology Section of Academia Sinica.

## From the institute of botany to the institute of microbiology

After the establishment of the People’s Republic of China, Fan-lan Tai participated in the preparation of the first national council of representatives of natural scientists, and promoted the revival and establishment of CSPP. In 1952, when the faculties were reorganized, Beijing Agricultural University was established, and in 1953, the CAS invited him to establish the Fungal Plant Pathology Laboratory of the Institute of Botany. In the same year, the CAS invited Fan-lan Tai to be the director of the laboratory. The laboratory was established on the campus of China Agricultural University and focused on plant disease control. He recruited five professors—Dafu Yu, Chuanguang Lin (林传光), Qiyi Shen (沈其益), Weifan Qiu (裘维蕃), and Huanru Wang (王焕如)—as part-time researchers, and later recruited several associate professors and lecturers to further enrich the research team of the laboratory (IMCAS, 2003). In the same year, CSPP was formally established, and Fan-lan Tai was elected as the president and editor-in-chief of *Acta Phytopathologica Sinica*. In 1955, he was elected as a member of the Department of Biology and Geology of CAS. At that time, the CAS proposed hiring Professor Shuqun Deng (邓叔群) from Shenyang Agricultural College to work in the laboratory. When some members expressed disagreement, Fan-lan Tai kept an open mind. From an academic point of view, he expressed his agreement with CAS, and promised to unify the department. The CAS invited him to be the deputy director of the laboratory.

Fan-lan Tai cultivated an international perspective, paying attention to academic exchange and cooperation. In 1954, he went to the Soviet Union with the Soviet May Day Observation Group of the Sino-Soviet Friendship Association to carry out an investigation in the field and strengthen international exchange. He also visited the Institute of Botany of the Academy of Sciences in Leningrad and the Department of Plant Protection of the Gimiriazev Agricultural College. He gave a report entitled “The Development of Phytopathology and Mycology in China,” and received a gift of many valuable books from Soviet phytopathologists (Ma, 1983). In 1955, together with Ying Ding, he participated in the fourth-anniversary celebration and scientific presentation and was awarded the title of corresponding academician of the Academy of Agricultural Sciences of the GDR (East Germany).

In December 1955, the China Association for Science and Technology, CAS, and several societies jointly held a conference to commemorate the centennial of the birth of Michurin. The organizers of the conference asked Fan-lan Tai to give the main report at the opening ceremony with a prepared script. Influenced by Lysenkoism, the one-sided propaganda of the Michurin doctrine, and the total rejection of theories of the Morganian School at that time, Fan-lan Tai, refused and said, “It is not appropriate to ask me to report. Besides, I am not a geneticist” (Bian, 2008). In 1956, a group of scientists from CAS joined the Communist Party of China, and Fan-lan Tai was the oldest member of this new group.

The laboratory was officially expanded to the Institute of Applied Mycology during the 12-Year Program for the Development of Science and Technology, and by 1954, the senior leaders and experts of CAS had already started to think about the establishment of the Institute of Microbiology. On 6 October 1955, the Department of Biology and Geology proposed “the idea of establishing a preparatory committee for the Institute of Microbiology” to the executive meeting of CAS. In 1958, the Department of Biology proposed merging the Institute of Applied Mycology with the Beijing Microbiology Research Laboratory and expanding it into the Institute of Microbiology. During this time, Fan-lan Tai patiently persuaded some older colleagues to ease their concerns. Afterwards, he unified the staff from the two units by determining the opinions for planning, determining the expansion of the use of microorganisms, and research on the prevention and control of harmful microorganisms as the main scope of activities, driving the disciplines of morphology, classification, ecology, physiology, and genetic variation of microorganisms (Wu et al., 2010). On 3 December 1958, the Institute of Microbiology was formally established, and Fan-lan Tai became its first director. After the establishment of the institute, he appealed to CAS several times because of the lack of laboratory space. He was undoubtedly an active academic thinker and willing to accept new things. When the new Microbial Physics Laboratory was established—soon after the merger of the two units—and needed to use isotopes, he was the first to agree to his colleagues’ proposal to establish a common isotope laboratory. Fan-lan Tai was also a person of integrity. He never signed his name when publishing research that he had not personally participated in, but stated in a footnote that he had supervised it, as a sign of responsibility (Qing, 2006).

## Fan-lan tai gave light and heat

In the 1960s, Fan-lan Tai started to compile the information he had collected over the decades and began to write the *Sylloge Fungorum Sinicorum*, which he insisted on writing in the dead of night, saying, “If I lose time during the day, I can make up for it at night. Work matters” (IMCAS, 2003). One month before his death, the first draft of more than 2 million words was completed. In 1979, the *Sylloge Fungorum Sinicorum* was published and became a masterpiece of Chinese mycology.

On 3 January 1973, Fan-lan Tai passed away. During his lifetime, he trained numerous mycologists and plant pathologists. His rigorous scientific attitude and noble character deserve to be studied and remembered by future generations.
